# Exploring Gender Differences within Forest Schools as a Physical Activity Intervention

**DOI:** 10.3390/children5100138

**Published:** 2018-09-26

**Authors:** Emilia Trapasso, Zoe Knowles, Lynne Boddy, Lisa Newson, Jo Sayers, Clare Austin

**Affiliations:** 1 Health Services Research, Institute of Psychology, Health and Society, University of Liverpool, Liverpool L3 5DA, UK; E.Trapasso@liverpool.ac.uk; 2 Physical Activity Exchange, Research Institute for Sport and Exercise Sciences, Liverpool John Moores University, Liverpool L3 2AT, UK; L.M.Boddy@ljmu.ac.uk (L.B.); C.L.Austin@2015.ljmu.ac.uk (C.A.); 3 Natural Sciences and Psychology, Research Centre for Brain and Behaviour, Liverpool John Moores University, Liverpool L3 5AF, UK; L.M.Newson@ljmu.ac.uk; 4 Community Development, The Mersey Forest Team, Risley Moss, Ordnance Avenue, Birchwood, Warrington WA3 6QX, UK; Jo.Sayers@merseyforest.org.uk

**Keywords:** Forest School, natural play, physical activity, children, mixed methodology, accelerometry, gender

## Abstract

This study investigated whether children engaged in more physical activity (PA) on school days that included Forest School (FS) sessions than a regular school day or a school day with a Physical Education (PE) lesson. How FS sessions influenced children’s general levels of PA and wellbeing was also explored across gender. A mixed-methods study followed a sample of 59 child participants aged 7 to 9 years old, from four primary schools, whilst taking part in twelve weekly FS sessions. Measures included the PA Questionnaire for Older Children and accelerometry data together with an individual Write and Draw task to inform focus groups. Children had significantly greater levels of light PA on a FS day and a PE school day compared to a regular school day and children reported feeling both happier and relaxed as a consequence of the intervention. From the qualitative data, boys and girls reported different likes of the FS interventions, whereas their dislikes of FS were comparable. Findings from this research provide evidence for such outdoor, nature-based learning within the school curriculum contributing to daily PA in children.

## 1. Introduction

Physical activity (PA) is associated with a wide range of health benefits for children and young people [1]. Recent reports state that in England, 24% of boys and 23% of girls aged 5–7 years and 78% of 5–15-year-olds are not meeting the Chief Medical Officers [2] recommendations of 60 min of moderate to vigorous PA (MVPA) per day, with only 23% of boys and 20% of girls meeting these recommendations on all 7 days of the week [3,4]. By the time they are aged 8 to 10 years, 26% of boys and only 16% of girls engage in 60 min per day of MVPA. Physical activity levels have been found to decline for both boys and girls throughout adolescence [5,6] and therefore effective early life-course intervention approaches to promote habitual PA and reduce sedentary behaviour (SB) are needed. More generally within the PA literature, previous research suggests that there are disparities for the PA levels of boys and girls, with boys being more likely to engage in MVPA than girls [4,7,8,9]. Pizarro et al. [10] reported that girls engaged in less MVPA during both school (24.7%) and leisure (18.1%) times, in comparison to their male counterparts (37% and 24.9%, respectively). 

School-based interventions have been suggested as one way to promote PA, with the school setting seen as an ideal environment for such interventions to take place [11,12,13]. A recent systematic review was conducted to explore the effectiveness of school-based interventions in promoting PA in children [14]. Based on an updated review of 44 studies, the review suggests that children exposed to school-based PA interventions are approximately three times more likely to engage in MVPA during the school day than those not exposed, whilst also finding a reduction in time spent watching television. Schools are known to influence children’s PA behaviours [15], however, there are some limitations to such school-based PA interventions. A more recent systematic review has explored evidence related to implementation of school-based PA models, finding many limitations to school-based PA interventions [16], including reporting of individual level interventions through the school setting, rather than a whole school approach to improving PA, with even fewer studies considering environmental or educational factors that may impact the outcome of the intervention. Time constraints were also a key influencing factor to implementation, reported in almost 80% of studies, suggesting that the preparation and delivery of the intervention competed with curriculum demands and resourcing for schools. Therefore, conducting an intervention that encourages PA through curriculum mapped activity may be a means by which to combat time constraints. Forest School (FS) is a regular, hands-on, child-led learning approach, set within a wooded environment, run by a trained school staff leader (or external FS leaders), which within a school setting is curriculum mapped and typically takes place during the school day; however, to date, it remains underexplored as to its contribution to daily PA. 

Forest Schools in the UK developed from the mid-1990s, in a direct response to a call for additional outdoor play, learning, and natural-based opportunities for children [17], initially following Bridgwater College’s observation and adaptation of early years children playing and learning outside in woodland in Denmark [18], and after the UK development of FS Leader training. The primary aims of FS have been described as promoting personal, social, and emotional development, focused on increasing self-esteem and positive learning dispositions, with secondary aims of providing opportunities for PA and contact with nature [19]. According to the Institute for Outdoor Learning Principles [20], FS should be a long-term process of frequent and regular sessions in a woodland or natural environment and take place regularly, with the same group of learners, over an extended period of time. Following basic safety rules, typical FS sessions encourage children to explore and play actively in a natural environment. Interventions are available for all ages and abilities, though particularly directed at children via education and daycare settings [21].

Benefits associated with participation in FS are restorative and wide reaching. Roe and Aspinall [22] noted that while all children benefitted from FS, children deemed as having ‘poor’ behaviour showed more positive changes in mood, ‘quieter’ children by virtue of special educational needs or ‘normally shy’ [23] reported more confidence post FS sessions, and children increased their abilities to interact socially and express themselves, with positive developments in communication [19,23,24,25,26]. Forest Schools has also been used successfully to foster natural play [27], whereby children explore and enjoy the natural environment through their freely chosen play activities [28]. This allows children to have increased interactions with the natural environment, as well as increasing knowledge, interest, and sensitivity towards nature [29,30]. Forest Schools engagement also provides improved knowledge, understanding, and respect for the natural environment, and increased ability to perceive and manage risk [19,29,31,32,33,34].

Only one study to date has evaluated the PA benefits of FS sessions [35], concluding that participants engaged in a greater amount of PA on a FS day (Light PA (LPA) = 34.26%, MVPA = 25.19%) compared to a regular school day (LPA = 18.98%, MVPA = 6.44%). Qualitative data reported that children appreciated the opportunity to be physically active outdoors, and differences between girls’ and boys’ motivation to be physically active was also lower on FS days, suggesting that FS was more inclusive, targeting and actively engaging both boys and girls. 

A limitation of the FS literature to date is that of small sample sizes, ranging from 10 to 24 participants [19,22,23,25,30], which thus offers low generalisability. The majority of FS research has been conducted to explore the associated benefits of FS, more so than the influences it can have on PA [19,23,24,26,27,32,36]. The dominance of qualitative and creative methodologies has, to date, offered rich, in-depth data from the participants [19,23,25,30,37] as well as the FS leaders [29,37]. Recent studies have used a Write and Draw technique to explore perceptions of PA [38,39], whereas others have adopted a focus group approach, a research technique considered appropriate with young children by having peers around them, which may help participants to feel more comfortable and express their thoughts [40]. The present study is unique, in terms of utilizing a combination of methods including objective data and, unlike previous research [35], a self-report measure to assess children’s habitual PA levels by gender rather than individual. Write and Draw techniques were also used to inform focus groups.

### Aims

The research aims were to investigate differences in PA levels between a FS day, a regular school day, a school day with a PE lesson, and a weekend day, whilst also assessing whether children’s general levels of PA would be higher as a result of FS sessions and exploring gender differences with regards to likes and dislikes of FS. The working hypothesis of this study was that PA levels will be higher on a FS day than a PE-school day and a school day without PE. 

## 2. Materials and Methods

### 2.1. Design

The present study used a mixed-methods approach adopting objective measures to investigate children’s PA with the primary outcome being PA levels measured through accelerometry data. Quantitative questionnaire data was collected at baseline and follow-up, objective seven-day PA monitoring, using accelerometers, was also completed mid-intervention. Qualitative explorations of FS experiences were conducted at follow-up, using both creative Write and Draw techniques and semi-structured focus groups, investigating gender differences in perceptions and experiences of FS, regular school lessons, and PE lessons; children’s likes and dislikes surrounding FS; and the impact of FS on children’s perceptions of wellbeing. The qualitative data provided context to the PA data collected. The methods chosen were age appropriate and offered a means by which the ‘the voice of the children’ could be elicited in the research. Data offered perceived intervention effects with regard to physical activity in FS sessions, wellbeing, and preferences on intervention design.

### 2.2. Participants

**Figure 1:**
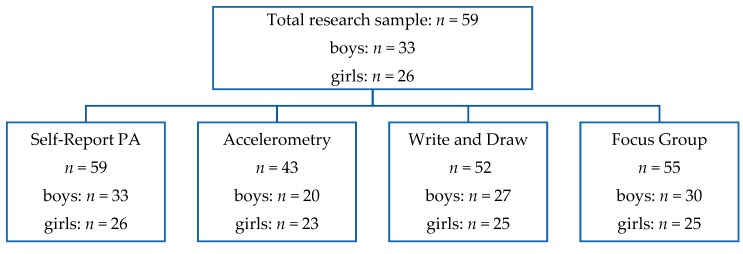
Diagram showing flow of participants throughout the research phases. PA: physical activity.

Fifty-nine children aged 7 to 9 years old (Mean = 8.17 years) were recruited from four primary schools in the North West of England (School A, *n* = 17, boys = 10, girls = 7; School B, *n* = 17, boys = 8, girls = 9; School C, *n* = 13, boys = 8, girls = 5; School D, *n* = 12, boys = 7, girls = 5). There was a random split of boys (*n* = 33) and girls (*n* = 26) across schools (see Figure 1 for flow of participants throughout the research phases). Sample size was based on the number of children involved in the externally delivered programme (FS) and the number returning informed consent (See Supplementary Materials); this was a service user evaluation based on those attending the FS intervention.

### 2.3. Settings

Participants took part in 12 weekly FS sessions. The FS sessions were delivered within each school’s well-established school woodlands, for 2 h per session, led by an external qualified FS Leader (with additional support in each school provided by trained FS leaders who were part of school staff). Forest Schools sessions were conducted in the spring/summer of 2014. Research data was collected independently by the research team. 

### 2.4. Procedure and Measures

#### 2.4.1. Self-Report Measure

The PA Questionnaire for Older Children (PAQ-C) [41] was used to assess children’s habitual PA before (prior to the first FS session) and upon completion of the FS sessions (at week 12). Physical Activity Questionnaire for Older Children is deemed a reliable and valid measure [42], where participants can score a range of 1–5. The PAQ-C was used in this study as a means of providing a summary PA score, and has been used extensively in previous children’s PA research [43,44].

#### 2.4.2. Anthropometrics

Anthropometric data collection was completed by the research team during the school day, at the mid-way point of the FS sessions (week 6). Stature and sitting stature were measured to the nearest 0.1 cm and body mass to the nearest 0.1 kg (Seca Ltd., Birmingham, UK) using standard techniques [45]. Using standard regression equations, somatic maturation (years to peak height velocity) was calculated for each participant [46]. Body mass index (BMI) and BMI Z-scores were calculated for each participant and weight status was classified according to international age and gender-specific cut-off points [47].

#### 2.4.3. Physical Activity Monitoring 

Uniaxial accelerometers (ActiGraph GT1M, MT1 Health Services, Pensacola, FL, USA) were provided to the children at the mid-point of the FS sessions (week 6) in order to objectively measure the children’s PA for seven consecutive days (which included a FS day). Accelerometers are valid and reliable for use with children [48] and have been used successfully in similar settings [35,49,50]. Participants were shown how to wear the monitors and when to remove them and put them back on. Children were instructed to wear their accelerometers on their right hip for seven consecutive days during waking hours. Information sheets were also given to parents and teachers. The monitors were set to record using 5-s epochs of data collection, in order to capture the sporadic nature of children’s PA [51]. Bouts of ≥20 min of consecutive zero counts (one-min spike tolerance) were used to define times when the monitor had been removed and were subtracted from daily wear time [52]. A valid day was defined as ≥9 h of monitor wear time on a week day and ≥8 h on a weekend day. Participants required any three valid days to be included within the analysis [53]. Physical activity data were classified into ST, LPA, moderate PA (MPA), and vigorous PA (VPA) using empirical cut-off points [54]. Moderate to vigorous PA was calculated as the sum of MPA and VPA. To promote compliance, a £10 National Book Token was provided for each child at the end of the monitoring period on return of the monitor. 

#### 2.4.4. Qualitative Measures—Write and Draw 

After 12 weeks of FS, Write and Draw activities were used to explore what children liked and disliked about their FS experiences. The task followed the same procedure as that of Knowles et al. [39] and was completed as a classroom activity. In summary, each child was given a two-sided sheet of paper, with the statement ‘What I like about FS is…’ on one side and ‘What I dislike about FS is…’ on the reverse. The statements each contained a large box underneath, which gave children the opportunity to Write and Draw an answer to these questions with materials supplied. 

#### 2.4.5. Qualitative Measures—Focus Groups

A total of 55 children (males: *n* = 30, females: *n* = 25) took part in 14 focus groups, with each having between two and five participants. Previous research suggested that having fewer than six children in a focus group would be optimal for retrieving good quality data from young children [55,56,57,58]. Open-ended questions explored the children’s perceptions of: whether FS sessions made them more active; how FS compared to a regular (non-FS) school day; how FS compared to PE lessons; and how FS influenced their wellbeing. Additional questions were added post analysis of the Write and Draw study that were designed to expand and further investigate themes revealed. The focus groups commenced in a quiet area within the school and lasted a mean of 38.29 min. A teaching assistant was present to comply with school safeguarding procedures. Focus groups were recorded using a Dictaphone (Olympus Europa, Hamburg, Germany).

### 2.5. Data Analysis 

#### 2.5.1. Self-Report Measure

A repeated measure analysis of covariance (ANCOVA) compared PA at baseline and at 12-week follow-up, measured by the PAQ-C [41].

#### 2.5.2. Physical Activity Monitoring 

A multivariate analysis of covariance (MANCOVA) was conducted to assess the differences between the average weekly total PA, LPA, MPA, VPA, and MVPA levels between boys and girls, and the differences in PA between FS days, regular school days (without PE lessons), PE lesson school days, and weekend days, while controlling for wear time.

#### 2.5.3. Qualitative Measures—Write and Draw

Thematic analysis (TA) [59] was used to explore children’s ‘likes’ and ‘dislikes’ regarding FS sessions. Thematic analisys included the following six analytical phases: (1) familiarisation with the data, (2) coding, (3) searching for themes, (4) reviewing themes, (5) defining and naming themes, and (6) writing up. The findings from the TA were subsequently demonstrated using pen profiles [39,40]. This approach has been previously used in qualitative work involving child participants in similar settings [38,39]. Pen profiles were used to represent overall analysis outcomes and provide an efficient representation of key themes and examples of verbatim data and frequency-related information, as opposed to all raw data themes recorded using more traditional TA procedures [60]. Quotations and pictures of the Write and Draw images were subsequently used to supplement the pen profiles and highlight emerging themes. To be included within the analytical process, drawings needed to be a legible representation of people, events, and/or places, labelling (using words) identifying factors (names, place, activity, etc.) and/or a denoted interaction or association. The following procedure and terminology were adopted to analyse the responses to the statements: ‘What I like about FS is…’ and ‘What I dislike about FS is…’ Responses to these statements were classified as a written ‘report’. When children reported more than one like or dislike, the reports were categorized to ‘marks’ in relation to a specific theme (i.e., activities, games, the environment). A ‘mark’ referred to where participant ‘reports’ were identifiable with a ‘theme.’ In most cases, one report identified more than one theme and subsequently more than one mark. For example, the report ‘I liked talking with my friends around the fire circle’ would require marks for more than one theme (both social interaction and activities). Outcomes from the analysis were used to inform the structure and questions asked in the subsequent focus groups. Write and Draw data specifically contributed to the literacy and context descriptions to shape the research questions.

#### 2.5.4. Qualitative Measures—Focus Groups

Audio files of the focus groups were transcribed verbatim and analysed using TA [59] to examine key themes and subthemes using the six phases of TA as described above. NVivo software (QSR NVivo, version 2.0.161, QSR InternationaL Ltd. Melbourne, Australia) assisted in the completion of data processing. Pen profiles were used to illustrate a composite of key themes from the data, deduced through a process of providing examples of verbatim data to illustrate themes. The extracted quotes or statements, made by the children, were self-definable and self-delimiting in the expression of a single recognisable aspect of their experiences. 

Within this study, credibility and transferability (qualitative equivalent of internal and external validity, respectively) [61] were demonstrated through verbatim transcription of focus group data and triangular consensus procedures for focus group and Write and Draw analysis. Triangulation of the analysis occurred through discussion of the pen profiles together with associated verbatim/illustrative material within the research team. The authors then critically questioned the analysis and interrogated the data independently, tracking the process in reverse from the pen profiles (or outcome) to the data source. This process continued until full consensus had been reached, limiting researcher bias from the analysis through an agreement of findings between authors. 

### 2.6. Ethical Considerations

The study received ethical approval from University Research Ethics Committee (UREC) prior to data collection (approval number 13/SPS/035). The children’s anonymity was upheld throughout this process; all personal and school names were replaced with pseudonyms during transcription. Head teachers provided gate keeper consent for school participation, and all children involved in the research returned informed parental consent and child assent forms prior to the study commencing.

## 3. Results

### 3.1. Physical Activity 

#### 3.1.1. Self-Report Measures 

Fifty-nine children completed the PAQ-C questionnaire. No significant differences in children’s self-reported PA levels were shown from baseline (Mean = 3.26, standard deviation (SD) = 0.76) to follow-up (Mean = 3.18, SD = 0.71) (*F*1, 57 = 1.93, *p* = 0.17). No differences were reported between the sexes (Table 1). 

#### 3.1.2. Accelerometry Results

Forty-three participants out of the fifty-nine involved in the study (*N* = 20 boys and 23 girls) met the accelerometer wear time inclusion criteria, resulting in a 72% compliance rate to the accelerometer protocol. There was a significant difference between boys’ and girls’ average weekly MVPA levels (*p* < 0.001). Boys averaged 75 min (SD = 20) MVPA per weekday compared to the girls, who averaged 55 min (SD = 16.1). Of the 43 participants, 63% (*n* = 27) of the sample (18 boys and 9 girls) achieved ≥60 min MVPA per day. Of the 43 participants that provided valid whole week data, only 36 (*N* = 17 boys, 19 girls) had valid data for a FS day, regular school day (without PE lessons), PE lesson school day, and weekend day, controlling for wear time. The adjusted mean, sedentary time, LPA, MPA, VPA, and MVPA, by days of the week are presented in Table 2. A repeated measures MANCOVA revealed that children had significantly greater levels of LPA (197.1 min/day vs. 173.7 min/day, *p* = 0.005) on a FS day compared to a regular non-PE school day, and significantly more LPA (198.9 min/day vs. 173.7 min/day, *p* = 0.001) on a PE day than on a regular non-PE school day. There were no significant differences between FS days, regular school days, PE lesson school days, and weekend days for sedentary behaviour, MPA, VPA, or MVPA. 

**Figure 2:**
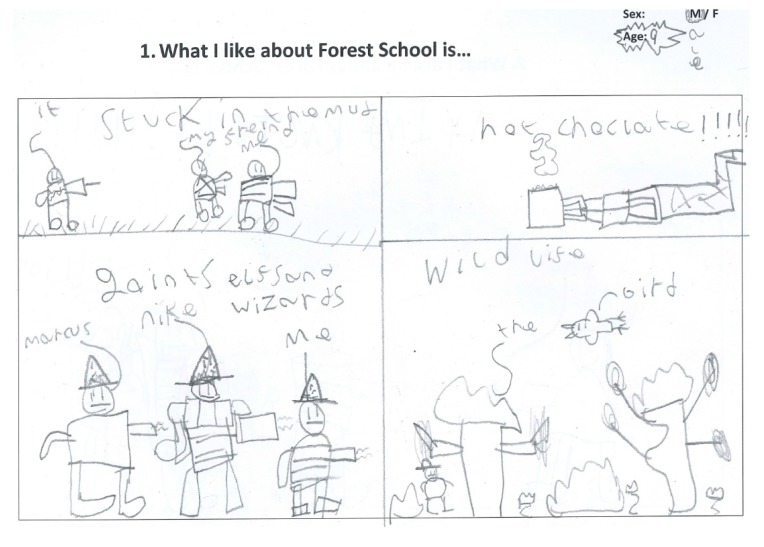
Drawing to illustrate boys’ likes.

**Figure 3:**
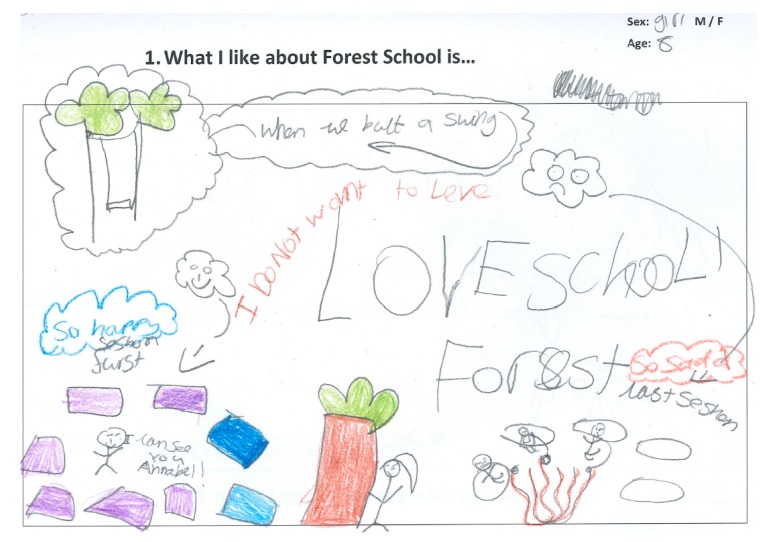
Drawing to illustrate girls’ likes.

**Figure 4:**
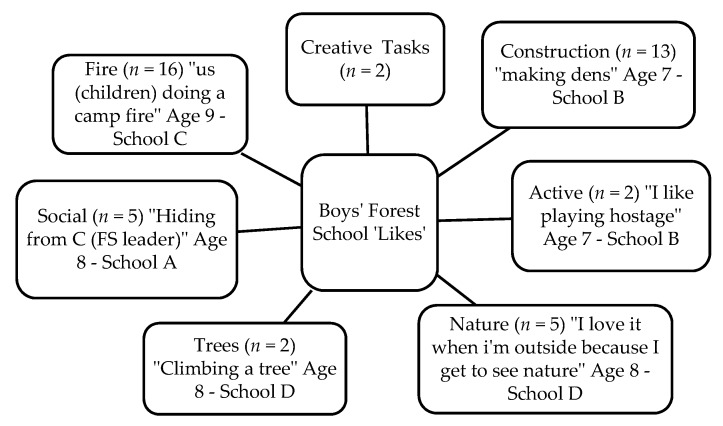
Pen profiles for boys’ answers to “What I like about Forest School is…?”.

**Figure 5:**
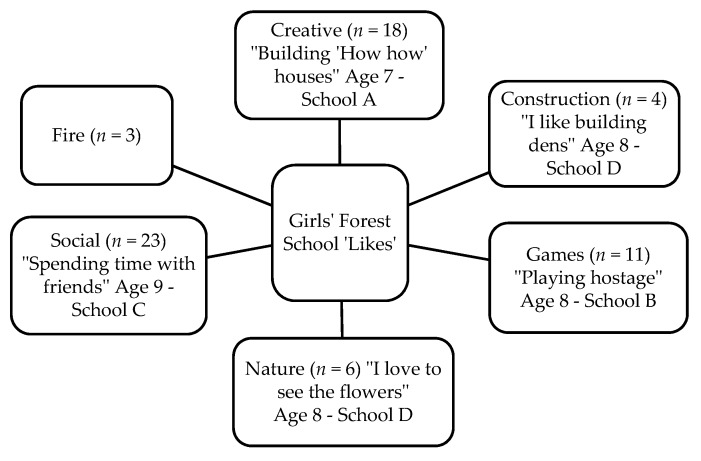
Pen profiles for girls’ answers to “What I like about Forest School is…?”.

**Figure 6:**
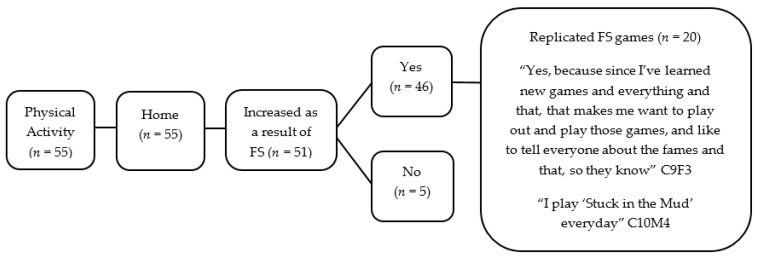
Pen profile demonstrating PA influenced by FS sessions.

**Figure 7:**
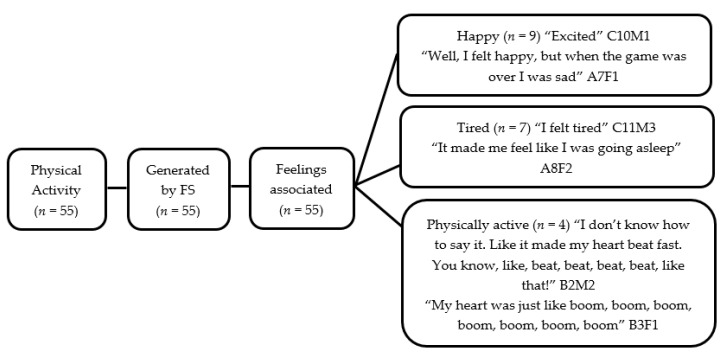
Pen profile demonstrating PA generated by FS and associated feelings.

Fifty-two children (27 boys and 25 girls) completed the Write and Daw activity (see Figure 2 and Figure 3). Figure 4, Figure 5, Figure 6 and Figure 7 illustrate the composite pen profiles for the statements ‘What I like about FS is…’ and ‘What I dislike about FS is…’ according to gender. In terms of responses to the statement ‘What I like about FS is…’, the fire (*n* = 16), active elements (*n* = 15), and construction activities (*n* = 13) were the most cited themes among boys (see Figure 2 and Figure 4), whereas social elements (*n* = 23), creative elements (*n* = 18), and games (*n* = 11) were the most commonly cited themes among girls (see Figure 3 and Figure 5). In response to the statement ‘What I dislike about FS is…’, the end of the sessions (*n* = 8 and *n* = 6) and the natural elements (*n* = 6 and *n* = 9) were the most cited themes among both boys and girls, respectively. 

### 3.2. Focus Group Data

#### 3.2.1. Physical Activity

When children were asked which elements of FS sessions involved PA, the majority of children referred to ‘chasing games’ played during the sessions (*n* = 41) (also see Figure 4). Children commented on how they felt physically active (*n* = 4) when playing these games and also how this made them feel happy (*n* = 9), for example, “I feel a bit more active, and I feel a bit more happy, because I keep moving, than when I’m just sitting down” (G13, P1, L246-247) (also see Figure 5). The majority of children rated their level of physical exertion as either ‘hard’ (*n* = 33) or ‘moderate’ (*n* = 13), with explanations such as; “I liked Hostage, because it made me very, very, very out of breath” (G1, P3, L89) and “I don’t know how to say it. Like it made my heart beat fast. You know, like beat, beat, beat, beat, like that” (G2, P1, L73-73) showing they could recognise and describe their physiological sensations when they were being active.

Children also discussed how they engaged in PA at home; the vast majority highlighted how they now played FS games outdoors (*n* = 43). Most of the children reported changes in their levels of PA as a result of FS. Many participants reported that they had replicated the games from their FS sessions (*n* = 20). “I’m going to do the same in Forest School … I got a skipping rope, and I have a tree, and I wrapped it round it, and I played Hostage” (G4, P4, L456-458) (also see Figure 6). Other participants discussed how since participating in FS they had now been involved in a lot more PA with their families at home, “I’ve been playing football more with my brother. I’ve been running round all over the place more” (G12, P1, L445-446). Thus, children reported that since FS had ended, they had been more physically active at home.

Whilst children did specifically recognise they were doing their PA outdoors, some did state that they also played indoors (*n* = 16), for example, “I don’t really play out, but I do go and play in mine with them” (G14, P1, L98). However, often this indoor play was linked to sedentary activities (*n* = 12), such as playing computer games, for example, “I always stay in on my PlayStation or on my tablet” (G5, P2, L96), with one individual explaining “I’m not really an exercisey boy like all the others” (G14, 92, L192), suggesting that if this participant played indoors they were typically not being physically active.

When asked how FS sessions had influenced the amount of PA they completed in their leisure time, the majority agreed that FS sessions had increased the amount of PA they participated in at home (*n* = 46).

It’s changed how much I play out, because I used to always just sit in my room, and I’d just sit there by myself, and then after I’ve started Forest School, it’s made me realise that instead of just sitting down I should be out there running around and everything. Because once I get fit I’d choose to just be adventurous and just play and be a tough girl at last (G9, P4, L314-318).

#### 3.2.2. Forest Schools and Classroom Comparisons: 

The majority of children said they preferred having their lessons outdoors as opposed to indoors (*n* = 51), for example, “Well, because outdoor you have hundreds of room, and indoor you don’t have lots of room” (G4, P1, L391-392), with many also stating that they preferred FS sessions to their regular classroom lessons (*n* = 23). Having increased opportunities to play (*n* = 9) was a reason why FS was seen as ‘better’ than classroom-based time. For example:

I think it was better in Forest School, because in class you have to sit down, and even though you get to play on Fridays, you get to play with the toys, it doesn’t mean you can just run around wild like you do in the forest when you get free time. And you mostly play great games in the forest, and you don’t play just in class like all the time, like when you were in class one. (G6, P3, L228-232).

Forest Schools allowed children to be active and have opportunities to play outdoors, whilst also being compliant to their school curriculum alongside nature-based learning activities. Children also reported that the behaviour of their peers was different in FS sessions, compared to when they were in a classroom environment (*n* = 18) agreeing that their peers were more helpful (*n* = 3), for example, “some people in our class are kind of a bit mean, but in Forest School they’re helpful sometimes, and they’re very good” (G4, P1, L380-381), with improved behaviour (*n* = 2), for example, “in class they’re a bit silly, but in the forest, because they know they need to keep safe, they listen to the rules” (G13, P1, L437-448), and some students were less shy (*n* = 2) in a FS environment, for example, “Well, I think people felt much better because they were having fun for once, and I’ve noticed a few shy people who were actually talking, like they weren’t shy” (G2, P2, L448-449)). Noticeably, the children did recognise FS as a place for learning, as well as a fun and playful experience.

#### 3.2.3. Forest Schools and Physical Education Lesson Comparisons

The main differences highlighted by children between their FS sessions and their PE lessons were that FS sessions were perceived by the children to be more active (*n* = 11), for example:

I feel different in PE because in PE I do get out of breath, but in Forest School I get out of breath, my face goes bright red, and I feel like I can’t run or anything, but in PE I can still run and that (G9, P3, L638-640).

Conversely, in PE lessons, the opportunity to play sport (*n* = 4) and the PE equipment available (*n* = 4) were highlighted as reasons PE lessons were seen as good (e.g., “because you go on the apparatus” (G1, P3, 556)). Some children did recognise how the FS environment was different (*n* = 7) to that of their PE lesson environment, and how this meant they could learn differently, for example:

I think they’re different because when it’s Forest School you get to see nature and all the birds, but when you’re inside you have to stay inside and just look out of the window to see nature, but it’s not actually touch nature (G13, P4, L477-479).

The children also felt they had more freedom (*n* = 5) in FS sessions in comparison to their PE lessons, for example, “I want school to be not fenced off, because it feels like it’s a prison, and you can’t get out anywhere” (G6, P4, L782-783). This may be attributed to the environment that FS takes place having fewer physical boundaries. When asked, some children stated that they preferred FS sessions to their PE lessons (*n* = 15), for example:

well, I think that PE is nowhere near as good as Forest School, because in PE it’s not very outdoory, where you can go and have a load of space and run around. There’s like climbing stuff or doing athletics, not like running around and having fun (G3, P4, L344-346).

Having free time (to choose what activities they wanted to engage in) during FS was considered a reason why the children preferred FS over PE lessons (*n* = 3) (e.g., “I like Forest School better, because you have free time” (G5, P2, L277)), with the environment in which it took place (*n* = 2) also being recognised as a reason FS is preferred over PE.

Well, Forest School we’re outside playing, and not being at school isn’t fun, but we’re having running round playing games. We have more plays and more chance, but then in the juniors we don’t have as much. And with Forest School, I guess you could say it’s sort of like a lesson, sort of helping teaching you. I think if I was in a forest now, and only had those things, I think I’d be a bit better at surviving. I think I’d be quite good at surviving from what I’ve learnt (G4, P3, L271-276).

#### 3.2.4. Wellbeing 

Children agreed that they enjoyed participating in FS sessions (*n* = 39) (also see Figure 7).

Excited. It was really fun, because I really enjoyed it, because you could play with sticks, and I love sticks. And you can make dens. It’s amazing! I just love it. And I think I’m speaking to everyone in the world here (G1, P1, L653-655).

**Figure 8:**
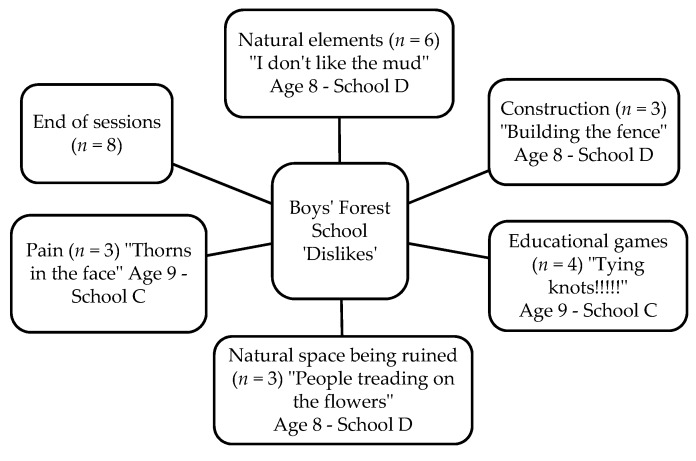
Pen profiles for boys’ answers to “What I dislike about Forest School is…?”.

**Figure 9:**
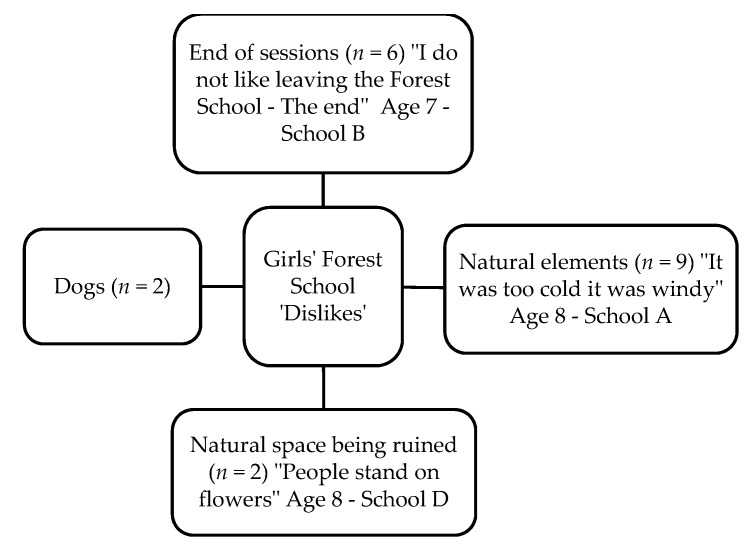
Pen profiles for girls’ answers to “What I dislike about Forest School is…?”.

The children highlighted opportunities for free play and creativity (*n* = 7) as a contributing factor to enjoyment and wellbeing. When the children were asked if FS sessions changed their mood and feelings; the majority of children agreed that the sessions improved their mood (*n* = 34) (for example, “It would make me feel better, because if I was annoyed with a person, it’d make me feel a bit more relaxed, and it’d make me happy” (G3, P4 L650-651) and another child explained “Because when you’re outside, you feel a little bit happy, then when you get inside you feel dead sad” (G7, P2, L524-525)). The children explained that FS had a positive impact by making them feel happier (*n* = 11), for example, “It would make me feel faster, it would make me feel stronger, it makes me happy, and it’s nice scenery, so it makes me very happy” (G11, P2, L517-518) (also see Figure 8 and Figure 9, where children noted that they disliked when FS ended). 

A final comment from one child after participating in FS demonstrated a perceived improvement for them in being outdoors.

Well, it’s had a big impact on my life, because I used to just sit inside and didn’t really like to go out, and when I’ve done Forest School it made me more used to being outside than inside (G4, P4, 215-217).

## 4. Discussion

This study used a mixed-methods approach to evaluate the PA benefits of FS sessions as a primary outcome. The primary aim of this study was to investigate the differences in PA levels between a FS day, a regular school day, a school day with a PE lesson, and a weekend day, with the working hypothesis that the children’s general levels of PA would be higher as a result of FS sessions. Secondary aims of this study were to use qualitative methods to explore whether FS sessions improved children’s mental wellbeing, and which elements of the FS sessions children liked or disliked. This study was unique, in terms of utilizing a combination of methods including objective data and, unlike previous research [35], a self-report measure to assess children’s habitual PA levels by gender rather than individual. 

### 4.1. Physical Activity

Objectively assessed PA data revealed that a relatively high proportion of the cohort met the >60 min/day recommendation (63% overall, 42% boys and 21% girls). The habitual PA levels described were higher than those reported in previous studies involving children in the UK [11,62]. Accelerometer findings demonstrated that children engaged in significantly more LPA on a FS day than on a regular school day and significantly more LPA on a PE lesson day than a regular school day. These findings are similar to previous research conducted with children and PA in FS [35], suggesting that FS may be a means by which children can accumulate PA during the school day. 

These findings were complimented by the qualitative focus group data surrounding PA gathered at follow-up. Many children reported sensations of physical exertion while participating in physically active games. Interestingly, accelerometer monitoring showed that PA levels in FS sessions were comparable to PE lessons; however, qualitative focus group findings suggest that FS sessions were reported by the children to be more physically active than PE lessons. Findings could be attributed to the physically active nature of the FS sessions combined with the outdoor environment where the sessions are held; indeed, children perceived themselves to be more active. Focus group data also supported the impact of FS sessions on children’s PA and the influence of FS sessions on their leisure time and general participation in outdoor play. Encouraging children to engage in FS interventions was shown to also expand to the children’s home environment, encouraging the children to be more active outdoors, although it could also be hypothesised to have a further influence on the children’s family and friends by encouraging them to also participate in such activities; as such, this type of intervention may have a wider impact on improving community PA levels through the concept of children being of agents of change [63,64,65].

The lack of significant differences in self-reported PA, measured by the PAQ-C [41], could suggest that FS sessions did not result in changes in PA behaviour. However, these findings could also be explained by the limitations associated with using questionnaires with this population and those associated with this questionnaire in particular [66], including issues with recall and social desirability bias. 

Light physical activity was higher on FS days and PE days when compared to a non-PE school days, which could be beneficial to health. While LPA is not included in the PA guidelines, evidence suggests that LPA provides health benefits through improvements to bone health, high-density lipoprotein-cholesterol, cardiorespiratory fitness, and diastolic blood pressure [67,68,69]. Children exceeded the recommended minimum threshold of 60 min of MVPA/day, so were an active group. Forest School days could make a meaningful contribution towards children meeting PA recommendations, though uniquely this is accumulated via curriculum time, in addition to, rather than in place of, other traditional PA opportunities (e.g., recess, PE lesson time, after school). Forest School activities also provided children with unique physical challenges that may be perceived differently to those offered during PE lessons [70]. 

### 4.2. Wellbeing 

Secondary outcomes, demonstrated through qualitative focus group data, included increased perceived wellbeing associated with taking part in FS sessions, consistent with previous studies [19,23,24,25,26]. Children also agreed that FS sessions positively influenced their mood by making them feel (using their words) happier. The combination of the increased opportunities for permitted free play, creativity, increased social interaction, and exercise could explain such reported improvements as well as the natural environment where sessions took place, which concurs with previous research [71,72]. Similar to previous studies [12,26], children also reported that peers who were perceived to have difficulties with behaviour, communication, or interaction behaved differently and more favourably in FS compared to their regular classroom lessons.

### 4.3. Gender Differences

Gender differences regarding children’s ‘likes’ of FS sessions are consistent with previous studies using Write and Draw data with a similar cohort [30]. Such findings prove useful to inform future gender-appropriate intervention design. Overall, boys and girls both enjoyed FS more than their normal school day and their PE lessons and felt that FS allowed them to be freer and more physically active. The girls and boys reported different preferences for FS content, which were consistent across the Write and Draw findings and the focus group data, with the boys liking fire, being active, and construction, whereas the girls liked social and creative elements as well as the games they played. However, gender dislikes were relatively similar, citing the same themes such as the sessions ending, and their dislike of some natural elements, such as being muddy and getting cold. Therefore, even though boys and girls found different elements of the FS enjoyable, this difference did not affect their levels of PA, and should not affect accumulations of PA. The FS leader should therefore be encouraged to make social games more active for the children, regardless of gender. 

### 4.4. Strengths and Limitations

This study is unique in terms of its aims, as it investigates PA as a primary outcome of FS, as well as considering children’s wellbeing and activity preferences within FS sessions. Several methodological considerations were regarded prior to data collection and analysis, ensuring they were appropriate for the participants and were effective in gathering the thoughts and perceptions related to the research topic. The strengths of the current study include the use of a mixed methodology, using quantitative, qualitative, and objective data collection techniques. Both the Write and Draw technique and the focus groups enabled rich in-depth data to be gathered from the child’s perspective, allowing an insight into their thoughts, beliefs, and experiences towards physical activity, respecting the expert knowledge of the participant [73]. Unlike previous research [35], this study also used a self-report measure to assess children’s habitual PA levels by gathering PA data from the child’s perspective. 

The mixed-methodology approach allowed for triangulation between the Write and Draw activity, children’s focus groups, and through using pen profiling together with associated verbatim or illustrative materials. The use of such an approach decreased the risk of misinterpreted views and potentially inaccurate data. Future research could, however, include a mapped link across self-reported PA data, accelerometry data, and qualitative findings from the Write and Draw techniques and the focus groups, allowing for comparisons to be made across all areas and encouraging the identification of whether those who reported higher PA through accelerometry data were also reporting having replicated FS session games at home, etc. 

The PAQ-C [41] results may have been influenced by poor recall, social desirability, and lack of precision. This instrument was developed to assess general levels of PA and does not therefore provide an accurate assessment of the frequency, intensity, and time of PA behaviours. These limitations could explain the lack of differences observed, and without objective PA measures taken at baseline and follow-up, the effect of FS sessions on children’s habitual PA could therefore not be assessed. Due to logistical constraints, we were unable to conduct objective assessments of PA pre- and post-intervention. Such measures would have provided useful information regarding any changes in habitual PA that were difficult to detect using the PAQ-C. 

## 5. Conclusions

Findings from this research provide evidence for curriculum mapped outdoor, nature-based learning within the school day having significantly greater levels of LPA on a FS day compared to a regular non-PE school day. Further, significantly more LPA was accumulated on a PE school day than on a regular non-PE school day, and children reported that they felt happier and more relaxed as a consequence of the intervention. The current study provides evidence that FS sessions are a valuable intervention by encouraging more LPA compared to regular non-PE school days. These benefits were also comparable to PE lesson school days, regardless of gender. FS sessions were also found to improve perceived mental wellbeing in children, with the children expressing that they felt happier and more relaxed as a result of the FS sessions. Notable gender differences were also demonstrated via Write and Draw data, consistent with previous studies and eliciting implications for FS curriculum design, showing that even though boys generally are more physically active than girls, they both showed increases in PA on FS days and both reported feeling happier during and after FS sessions.

## Figures and Tables

**Table 1:** Participant characteristics.

	Total Mean (SD)	Male Mean (SD)	Female Mean (SD)
**Height (cm)**	130.62 (6.77)	130.57 (7.26)	130.68 (6.23)
**Weight (kg)**	32.75 (8.83)	32.42 (10.06)	33.15 (7.15)
**BMI**	18.94 (3.53)	18.69 (3.86)	19.26 (3.09)

BMI: body mass index, SD: standard deviation.

**Table 2:** Mean (standard error in parentheses) sedentary behaviour, light PA, moderate PA, vigorous PA, and total PA for Forest School (FS) days, Physical Education (PE) lesson days, weekend days, and regular school days adjusted for accelerometer wear time (*n* = 36, boys: *n* = 17, girls: *n* = 19).

	Forest School Day	PE Lesson Day	Weekend Day	Regular School Day
**Sedentary behaviour** (min/day)	414.9 (8.6)	417 (7.8)	420.3 (8.4)	430.1 (7.3)
**Light PA** (min/day)	* 197.1 (5.3)	** 198.9 (4.5)	191.2 (5.2)	*, ** 173.7 (4.5)
**Moderate PA** (min/day)	40.8 (2.4)	37.8 (2.2)	39.8 (2.4)	34.8 (2.1)
**Vigorous PA** (min/day)	29.6 (2.5)	28.6 (2.3)	29.6 (2.5)	27.2 (2.1)
**Moderate to vigorous PA** (min/day)	70.3 (4.4)	66.4 (4.1)	69.5 (4.4)	62.3 (3.8)
**Total PA** (min/day)	267.2 (8.1)	265.1 (7.4)	261.4 (8)	249.1 (7)

* *p* = 0.005 (FS day > Regular School day). ** *p* = 0.001 (PE day > Regular School day).

## Supplementary Materials

The following are available online at http://www.mdpi.com/2227-9067/5/10/138/s1.
