# Supplementary material for: Exploring Gender Differences within Forest Schools as a Physical Activity Intervention
Source: Children (Basel). 2018 Sep 26;5(10):138. doi: 10.3390/children5100138 (PMC6211073; doi:10.3390/children5100138)
Supplement: Supplementary file 1 [file children-05-00138-s001.pdf]

Appendix 1.

LIVERPOOL JOHN MOORES UNIVERSITY
PARENTAL CONSENT FORM

Natural Health Service in the Mersey Forest: Forest Schools

Dr Zoe Knowles, Research Institute for Sport and Exercise Sciences

Please tick/cross the relevant boxes below:

1. I confirm that I have read and understand the information provided for the above study. I have had the opportunity to consider the information, ask questions and have had these answered satisfactorily. [1]
2. I understand that my child's participation in the research is voluntary and that I am free to withdraw him/her at any time, without giving a reason and that this will not affect my legal rights. [2]
3. I understand that any personal information collected during the study will be anonymised and remain confidential. [3]
4. I give permission for photographs to be taken of my child during the project, which may be used for subsequent academic/promotional purposes associated with The Mersey Forest. [4]
5. I am aware that children will be weighed and measured and these results will remain confidential [5]
6. I give permission for my child to wear an accelerometer to record their physical activity [6]
7. I agree for my child to take part in the above study. [7]

Name of Child ____

Name of Parent/Guardian: ____

Signature: ____________________________________ Date: _________________

Name of Researcher	Date	Signature
Name of Person taking consent<br>(if different from researcher)	Date	Signature

NOTE: When completed 1 copy for participant and 1 copy for researcher

Parent/Guardian Contact Sheet

Please only complete this section if you are interested in receiving further information, receiving information about research findings and about participating in future activities The Mersey Forest has to offer.

Name of Parent/Guardian: ______________________________

Name of Child: ______________________________

Address:

____

____

____

____

Contact telephone number: ______________________________

E-mail address (if applicable): ______________________________

Please state what your preferred method of contact is: ______________________________

Note: The details provided here **will not be shared with any organisations outside the study.** The information will be used to inform you of family activities, research findings in this project and to send out involvement vouchers.

liverpool JMU

THE MERSEY FOREST more from trees

NOTE: When completed 1 copy for participant and 1 copy for researcher

Appendix 2.

LIVERPOOL JOHN MOORES UNIVERSITY ASSENT FORM FOR CHILDREN / OTHER DEPENDENTS

(to be completed by the child and their parent/guardian)

Natural Health Service in the Mersey Forest: Forest Schools

Dr Zoe Knowles, Research Institute for Sport and Exercise Sciences

Child (or if unable, parent/guardian on their behalf) / young person to circle all they agree with:

1. Have you read (or had read to you) information about this project? Yes/No
2. Has somebody else explained this project to you? Yes/No
3. Do you understand what this project is about? Yes/No
4. Have you asked all the questions you want? Yes/No
5. Have you had your questions answered in a way you understand? Yes/No
6. Do you understand it's OK to stop taking part at any time? Yes/No
7. Are you happy to take part? Yes/No

If any answers are 'no' or you **don't** want to take part, don't sign your name!

If you **do** want to take part, you can write your name below

Your name ____________________________

Date ____________________________

Your parent or guardian must write their name here if they are happy for you to do the project.

Print Name ____________________________

Sign ____________________________

Date ____________________________

The researcher who explained this project to you needs to sign too.

Print Name

NOTE: When completed 1 copy for participant and 1 copy for researcher
